# Family with sequence similarity 13 member A mediates TGF-β1-induced EMT in small airway epithelium of patients with chronic obstructive pulmonary disease

**DOI:** 10.1186/s12931-021-01783-z

**Published:** 2021-07-01

**Authors:** Jinyuan Zhu, Faxuan Wang, Xueyan Feng, Beibei Li, Liqiong Ma, Jin Zhang

**Affiliations:** 1 Department of Critical Care Medicine, General Hospital of Ningxia Medical University, Yinchuan, 750004 People’s Republic of China; 2 School of Public Health and Management, Ningxia Medical University, Yinchuan, 750004 People’s Republic of China; 3 Ningxia Medical University, Yinchuan, 750004 People’s Republic of China; 4 Department of Pathology, General Hospital of Ningxia Medical University, Yinchuan, 750004 People’s Republic of China; 5 Department of Respiratory and Critical Care Medicine, General Hospital of Ningxia Medical University, 804 Shengli South Street, Xingqing District, Yinchuan, 750004 People’s Republic of China

**Keywords:** FAM13A, TGF-β, Epithelial–mesenchymal transition, Chronic obstructive pulmonary disease, Small airway epithelium

## Abstract

**Background:**

To explore the role of family with sequence similarity 13 member A (FAM13A) in TGF-β1-induced EMT in the small airway epithelium of patients with chronic obstructive pulmonary disease (COPD).

**Methods:**

Small airway wall thickness and protein levels of airway remodeling markers, EMT markers, TGF-β1, and FAM13A were measured in lung tissue samples from COPD and non-COPD patients. The correlations of FAM13A expression with COPD severity and EMT marker expression were evaluated. Gain- and loss-of-function assays were performed to explore the functions of FAM13A in cell proliferation, motility, and TGF-β1-induced EMT marker alterations in human bronchial epithelial cell line BEAS-2B.

**Results:**

Independent of smoking status, lung tissue samples from COPD patients exhibited significantly increased small airway thickness and collagen fiber deposition, along with enhanced protein levels of remodeling markers (collagen I, fibronectin, and MMP-9), mesenchymal markers (α-SMA, vimentin, and N-cadherin), TGF-β1, and FAM13A, compared with those from non-COPD patients. FAM13A expression negatively correlated with FEV_1_% and PO_2_ in COPD patients. In small airway epithelium, FAM13A expression negatively correlated with E-cadherin protein levels and positively correlated with vimentin protein levels. In BEAS-2B cells, TGF-β1 dose-dependently upregulated FAM13A protein levels. FAM13A overexpression significantly promoted cell proliferation and motility in BEAS-2B cells, whereas FAM13A silencing showed contrasting results. Furthermore, FAM13A knockdown partially reversed TGF-β1-induced EMT marker protein alterations in BEAS-2B cells.

**Conclusions:**

FAM13A upregulation is associated with TGF-β1-induced EMT in the small airway epithelium of COPD patients independent of smoking status, serving as a potential therapeutic target for anti-EMT therapy in COPD.

**Supplementary Information:**

The online version contains supplementary material available at 10.1186/s12931-021-01783-z.

## Background

Chronic obstructive pulmonary disease (COPD) is a progressive and partially reversible disease characterized by airflow obstruction resulting from a chronic inflammatory response of the airways and lungs to noxious particles or gases [1]. The prevalence of COPD ranges from 8.80 to 14.53% among different countries [2], and COPD accounts for over 2 million deaths worldwide every year [3]. It has been predicted that COPD will rise from the fourth to the third leading cause of global death from 2013 to 2020 [4]. Therefore, it is important to understand the underlying mechanisms of COPD development and identify novel therapeutic targets for COPD treatment.

Small airways (< 2 mm internal diameter) are the major sites of airflow obstruction in patients with COPD [5]. Small airway remodeling occurs in the early stage of COPD and largely contributes to airway obstruction in the disease [6]. Peribronchiolar fibrosis, airway thickening resulting from the excess amount of extracellular matrix (ECM), loss of alveolar attachments, and inflammatory cell infiltration are considered markers of small airway remodeling in COPD [7]. Recent studies have suggested epithelial–mesenchymal transition (EMT), the conversion of adherent epithelial cells into migratory mesenchymal cells, as a core pathological factor in airway remodeling during COPD development [8, 9]. The airway epithelium from COPD patient exhibits persistent activation of EMT, with decreased protein levels of epithelial markers such as E-cadherin and zona occludence-1 (ZO-1) and increased protein levels of mesenchymal markers, including N-cadherin, vimentin, and α-smooth muscle actin (α-SMA) [10–12]. Investigators have demonstrated that transforming growth factor-beta 1 (TGF-β1) expression is upregulated in the airway epithelium of COPD patients and that TGF-β1 induces EMT in the bronchial epithelial cells [13, 14]. However, the underlying mechanism underlying the regulation of TGF-β1 in EMT during COPD small airway remodeling is not fully understood.

Although smoking is the primary risk factor for COPD, genetic variation also contributes to the COPD genesis [15]. Studies have reported that variants in the family with sequence similarity 13 member A (FAM13A) are associated with chronic lung diseases, including COPD, asthma, lung cancer, and pulmonary fibrosis [16–19]. We previously demonstrated that a genetic variant in FAM13A correlates with the increased risk of COPD in never-smokers [20]. This suggests that FAM13A might be involved in the genesis and development of COPD independent of smoking status. FAM13A protein contains a rho GTPase activating protein domain that inactivates EMT-associated GTPases with converting GTP to GDP [21]. FAM13A has been shown to regulate EMT in human bronchial epithelial cells and pulmonary artery endothelial cells [19, 22]. Based on these findings, we hypothesized that FAM13A might participate in TGF-β1-induced EMT during small airway remodeling in COPD.

To test our hypothesis, we measured the FAM13A expression in the small airway epithelium of lung tissue samples from COPD and non-COPD patients. We evaluated the correlations of FAM13A expression with COPD severity and EMT marker protein levels. We also investigated the roles of FAM13A in cell proliferation and motility and TGFβ1-induced EMT in human bronchial epithelial cell line BEAS-2B. Our findings identify FAM13A as a novel mediator of TGFβ1-induced EMT in the small airways, providing FAM13A as a potential therapeutic target for COPD treatment.

## Methods

### Patients and specimens

A total of 69 patients were recruited in the Department of Thoracic Surgery, General Hospital of Ningxia Medical University between January 2018 and December 2019. These patients were 35 patients with lung adenocarcinoma, 17 patients with lung squamous cell carcinoma, 12 patients with benign lung nodules, and 5 patients with bullous emphysema. Before the study, all the patients with lung adenocarcinoma, lung squamous cell carcinoma, and benign lung nodules have undergone lobectomy. The patients with bullous emphysema have undergone lung volume reduction surgery.

The inclusion criteria were: (1) lung cancer patients presenting a single nodule without lung metastasis in preoperative CT scan; (2) COPD patients with a post-bronchodilator forced expiratory volume in 1 s (FEV_1_)/forced volume vital capacity (FVC) ratio < 70%; (3) smokers (smoking history > 5 years and quitting smoking < 2 years) with a post-bronchodilator FEV_1_/FVC ratio > 70%; (4) never-smokers with a post-bronchodilator FEV_1_/FVC ratio > 70%; (5) pulmonary function testing was performed within 3 months before surgery.

The exclusion criteria were: (1) chronic lung diseases, such as asthma, tuberculosis, pneumonia, and interstitial lung disease; (2) acute COPD or discontinuing treatment < 2 months; (3) declining research participation. The patients were divided into non-smokers without COPD (n = 22), smokers without COPD (n = 23), non-smokers with COPD (n = 10), and smokers with COPD (n = 14) groups. The clinical characteristics of the patients were summarized in Table 1.

**Table 1:** Clinical characteristics of patients

Characteristics	Non-smokers without COPD (n = 22)	Smokers without COPD (n = 23)	Non-smokers with COPD (n = 10)	Smokers with COPD (n = 14)	$\chi ^{2}$*/t/F*	*P-*value
Gender					$\chi ^{2}$ = 1.828	0.609
Male	10	14	4	8		
Female	12	9	6	6		
Age (years $\bar{x} \pm s$)	55.2 ± 12.1	56.8 ± 15.3	57.1 ± 13.4	56.1 ± 15.3	*F* = 1.745	0.424
Smoking index (package year) ($\bar{x} \pm s$)	–	36 ± 14	–	45 ± 13	*t* = 1.210	0.252
FEV_1_/FVC (%)	77.3 ± 4.3	75.8 ± 2.2	65.6 ± 2.6*^#^	62.9 ± 3.9*^#^	*F* = 44.745	< 0.001
FEV_1_%pre (%)	94.3 ± 11.5	93.1 ± 7.6	63.4 ± 7.1*^#^	64.1 ± 5.8*^#^	*F* = 35.451	< 0.001
PO_2_ (mmHg)	80.12 ± 16.34	79.82 ± 17.64	60.31 ± 11.21*^#^	58.87 ± 16.34*^#^	*F* = 38.65	< 0.001
PCO_2_ (mmHg)	37.34 ± 12.35	38.87 ± 14.15	41.64 ± 15.85*^#^	42.59 ± 19.05*^#^	*F* = 31.87	< 0.05
BMI (kg/m^2^)	24.31 ± 3.30	24.43 ± 2.81	22.5 ± 2.23*^#^	*22.38* ± 2.41*^#^	*F* = 30.124	< 0.05
Disease constitution					-	0.270
Lung adenocarcinoma	12(54.5%)	11(47.8%)	5(50.0%)	7(53.9%)		
Lung squamous cell carcinoma	6(27.3%)	7(30.4%)	2(20.0%)	2(15.4%)		
Lung nodules	4(18.2%)	5(21.8%)	1(10.0%)	1(7.7%)		
Bullous emphysema	0(0.0%)	0(0.0%)	2(20.0%)	3(23%)		

*P < 0.05 vs. the non-smokers without COPD group

^#^*P* < 0.05 vs. the smokers without COPD group

Lung tissue samples (5 cm × 5 cm; > 5 cm from the tumor margin) were obtained from the patients during lobectomy or lung volume reduction surgery. All tissue samples were immediately processed upon surgical removal. After fixing with 4% paraformaldehyde, the tissue samples were dehydrated in a graded ethanol series, paraffin-embedded, and cut into 4-µm-thick sections. This study was approved by the Ethics Committee of General Hospital of Ningxia Medical University. Written consent was obtained from all patients.

### Cell culture and TGF-β1 stimulation

BEAS-2B cells were provided by Professor Fuqiang Wen at West China Hospital, Sichuan University (Chengdu, Sichuan, China). The cells were maintained in DMEM/F12 (1:1; Gibco, Thermo Fisher Scientific, Waltham, MA, USA) supplemented with 10% fetal bovine serum (FBS; Gibco) and 1% penicillin/streptomycin (Solarbio, Beijing, China) in a humidified atmosphere of 5% CO_2_ at 37 °C. TGF-β1 in various concentrations (PepproTech, Rocky Hill, NJ, USA) was used to treat BEAS-2B cells (see concentrations in later methods).

### Construction of lentiviral expression vectors and transfection

For the overexpression of FAM13A, we collected RNA samples from clinical COPD patients and examined the mRNA expression of the five transcripts of FAM13A according to entries in NCBI and identified the mRNA level of the FAM13A transcript (NM_001015045.2) as higher than those of the other four transcripts. Therefore, the coding sequence of human FAM13A (NM_001015045.2) was subsequently cloned into a lentiviral vector GV492 (Genechem, Shanghai, China). Double-stranded DNA-coding small hairpin RNA (shRNA) for FAM13A (5′-ACACCAAACAGCAGAGAAA-3′, synthesized by Genechem) was cloned into a lentiviral vector GV248 (Genechem). Lentivirus was packaged in HEK293T cells. For lentiviral transduction, BEAS-2B cells were transduced with 15–multiplicity of infection of lentiviral vectors expressing FAM13A (LV-FAM13A) or shRNA against FAM13A (shFAM13A) for 48–72 h (Additional file 1: Fig. S1). BEAS-2B cells transfected with the empty GV492 vector and the empty GV248 vector were used as the negative control for overexpression and knockdown, respectively.

### Measurement of small airway wall thickness

The deparaffined sections were stained with hematoxylin and eosin using standard methods for histological examination. The absence of tumor tissue was confirmed by two different pathologists who determined more accurately by observing the morphology of the cells in the HE-stained sections. The airway wall thickness was measured in 3–5 small airways with a basement membrane perimeter < 1000 μm in each section, as previously described [23]. Image-pro Plus (IPP) 6.0 software (Media Cybernetics, Bethesda, MA, USA) conferred quantification of stained samples, which can quantify a length directly, quantify an area by outlining the staining and calculation, or quantify the staining by weighing the optical density and calculation. Using IPP 6.0, the length of the basement membrane (Pbm), the area defined by the basement membrane (Abm), and the area defined by the total adventitial perimeter (Ao) were all quantified. The calculation formula used was Thickness = total bronchial wall area (WAt)/Pbm, which was calculated as Ao − Abm.

### Measurement of collagen area

The collagen fibers in the lung tissue samples from patients were stained using a Masson’s trichrome staining kit (Solarbio, Beijing, China) following the manufacturer’s instruction. The Blue-stained collagen fibers were observed under an Olympus microscope (Olympus, Japan). The percentage of the collagen area was measured as previously described [24]. Briefly, the total collagen area (stained by Masson's trichrome) was quantified using the IPP6.0 software. The calculation formula for the percentage of the collagen area was: Collagen area (%) = Area/Pbm.

### Immunohistochemical (IHC) staining

The protein levels of small airway remodeling markers and EMT markers were determined by immunohistochemical staining. Briefly, the paraffin-embedded sections were dewaxed in xylene and dehydrated in ethanol, followed by incubation with 3% H_2_O_2_ for 10 min. After antigen retrieval, the sections were blocked with 10% normal goat serum, then incubated with anti-FAM13A (1:800; HPA038109, Sigma, Dorset, UK), anti-fibronectin (1:500; ab2413, Abcam, Cambridge, UK), anti-E-cadherin (1:400; ab76055, Abcam), anti-α-SMA (1:500; ab7817, Abcam), anti-ZO-1 (1:200; ab216880, Abcam), anti-MMP9 (1:500; ab76003, Abcam), anti-N-cadherin (1:500; ab18203, Abcam), anti-collagen I (1:500; ab34710, Abcam), or anti-TGF-β1 (1:1000; ab27969, Abcam) antibody. Detection of the antigen–antibody complex was performed using a secondary antibody and a DAB detection kit. All the secondary antibodies and the DAB detection kit were purchased from Beijing Zhong Shan-Golden Bridge Biological Technology (Beijing, China). The results were examined and photographed under an Olympus microscope (Olympus, Japan) at a magnification of × 400. Except for vimentin, the expression of target proteins was expressed as the average optical density (AOD) of the positive staining in 5 randomly selected fields as previously described [25]. Briefly, the integrated optical density (IOD) and the total area of staining (Area) were quantified by IPP6.0. The calculation formula of AOD was: AOD = IOD/Area. Vimentin expression was calculated as vimentin-positive cell numbers/Pbm, as previously described [8]. The former was manually counted while Pbm was quantified using IPP 6.0 as already described.

### Immunocytochemical and immunofluorescent staining

FMA13A expression in response to TGF-β1 stimulation was detected by immunocytochemical and immunofluorescent staining. Briefly, BEAS-2B cells were seeded on a coverslip in a 24-well plate at a density of 1 × 10^4^ cells/well and incubate overnight. Cells were treated with TGF-β1 (10 ng/mL; PepproTech) for 48 h. For immunocytochemical staining, cells were fixed with 4% paraformaldehyde for 20 min at room temperature. After blocking with 10% normal goat serum for 20 min, cells were incubated with anti-FAM13A (1:100, Sigma-Aldrich, St. Louis, MO, USA) at 4 °C overnight, followed by incubation with HRP-conjugated secondary antibody (Beijing Zhong Shan-Golden Bridge Biological Technology) for 20 min at room temperature and for 10 min counter-staining by using haematin and hydrochloric acid 1% in 70% alcohol. After rinsing with tap water, the slides were dried and sealed with neutral resin. Images were acquired under an Olympus microscope.

For immunofluorescent staining, cells were fixed with 4% paraformaldehyde for 20 min at room temperature. For FAM13A detection, after blocking with 5% bovine serum albumin in phosphate-buffered saline (PBS), cells were incubated with anti-FAM13A at 4 °C overnight with a dilution of 1:100, followed by 3 washes with PBS. The cells were then incubated with a FITC-conjugated secondary antibody (Goat Anti-Rabbit IgG H&L, ab150081, Abcam) in the dark for 1 h at room temperature. For filamentous actin (F-actin) detection, after blocking with 1% bovine serum albumin, cells were incubated with phalloidin-iFluor 647 Conjugate (40734ES75, Yeasen biotech, Shanghai, China) in the dark for 30 min at room temperature. The fluorescence was visualized using an Olympus fluorescence microscope.

### Bromodeoxyuridine (BrdU) staining

A total of 10 mg BrdU powder (ab142567, Beijing Zhong Shan-Golden Bridge Biological Technology) was dissolved in 10 mL pure water (injection-grade) to make a BrdU solution at a stock concentration of 1.0 mg/mL were stored at 4 ℃ in the dark. BEAS-2B cells were prepared in a 12-well plate and cultured, as previously described, until the cell density of 50%. The BrdU stock solution was further diluted to a final concentration of 0.03 μg/mL and filled into the wells were incubated at 37 ℃ for 4 h before BrdU was removed, followed by washing with PBS for three times. The cells were fixed with 4% paraformaldehyde for 30 min and washed using PBS for three times (5 min each time). Next, HCL (2 mol/L) was applied to the cells for 30 min at room temperature to dissolve DNA. After washing with PBS for three times (5 min each time), the cells were incubated with the blocked membranes in 5% BSA for 1 h at room temperature. The cells were subsequently incubated overnight with the primary antibody (ab6326, 1:200, Abcam) at 4 ℃ and washed with PBS for three times. This was followed by incubation with the secondary antibody (1:200, A-21209, Invitrogen, USA) for 2 h and washing with PBS for three times. The 4′, 6-diamidino-2-phenylindole (DAPI, 1:500, Sigma, USA) was then added to stand for 2 min before PBS washing for three times. Lastly, a fluorescent quenching agent (Solarbio, China) was added for photo-taken.

### Cell migration and invasion assays

Cell migration and invasion were measured with the wound healing assay and Transwell chamber assay, respectively. For the wound healing assay, BEAS-2B cells transfected with LV-FAM13A, shFAM13A, or corresponding negative control were seeded into a 6-well plate at a density of 1 × 10^5^ cells per well and cultured overnight. A 200 µL micropipette tip was used to generate a 2 mm-wide scratch line in the cell monolayer. Cells were allowed to migrate for 24 h. Images were captured under an inverted light microscope (Olympus, Japan).

For the invasion assay, BEAS-2B cells transfected with LV-FAM13A, shFAM13A, or corresponding negative control were loaded into a Matrigel-coated upper chamber (Corning, Corning, NY, USA) filled with serum-free DMEM. The lower chamber was filled with 500 μL DMEM containing 20% FBS. After incubation at 37 °C for 48 h, nonmigrating or non-invading cells remaining in the upper chamber were removed with a cotton swab. The migrating or invading cells adhering to the lower surface were fixed and stained with crystal violet (0.1%). The stained cells were counted in 3–5 randomly selected fields under an inverted light microscope (Olympus) at × 200 magnification. Images were acquired using an Olympus camera (Olympus, Japan).

### Cell proliferation and apoptosis analyses

Cell proliferation and apoptosis were measured with flow cytometry analysis. For cell proliferation analysis, BEAS-2B cells (1–5 × 10^5^/mL) were harvested at 48 h after transfection and fixed with 80% methanol for 5 min. Cells were washed with PBS and fixed with 4% paraformaldehyde for 20 min at room temperature, followed by penetration with 0.1% Triton X-100. After 30 min of blocking with 10% normal goat serum (Solarbio), cells were incubated with anti-Ki67 (1:100; ab16667; Abcam) for 30 min at 22 ℃. Cells were then incubated with secondary antibody (1:500, Goat Anti-Rabbit IgG H&L ab150083; Abcam) for 30 min at 22 ℃, followed by resuspension in PBS and flow cytometry analysis on a BD Accuri flow cytometer (Becton Dickinson, San Jose, CA, USA).

Cell apoptosis was measured using an Annexin V-PE/7-AAD apoptosis kit (Liankebio, Hangzhou, Zhejiang, China) following the manufacturer’s instruction. Briefly, BEAS-2B cells (1–3 × 10^6^/mL) were harvested at 48 h after transfection. Cells were digested with enzymes free of ethylenediaminetetraacetic acid (EDTA) to prepare into single-cell suspensions. Following counting, the cells were re-suspended with Annexin binding buffer (containing HEPES buffer, calcium chloride, and Ca-free), supplemented with Annexin V reagents and PE in the kit. After washing with ice-cold PBS, cells were reconstituted in the binding buffer and stained with Annexin V-PE/7-AAD, followed by flow cytometry analysis on a BD Accuri flow cytometer.

### Quantitative real-time PCR (qRT-PCR)

FAM13A mRNA expression in BEAS-2B cells after transfection was determined using qRT-PCR. Cells were seeded in a 6-well plate at a density of 2 × 10^5^ cells/well and grown overnight. Cells were transfected with LV-FAM13A, shFAM13A, or corresponding negative control for 48 h. Total RNA was isolated using Trizol reagent (Abcom, USA) following the manufacturer’s instruction. cDNA was synthesized using a reverse transcription kit (Takara, Japan). qPCR was performed using SYBR Green (Takara) and gene-specific primers (FAM13A, forward: 5′-AAGTCTGCCTCATTGGCTGTGG-3′, reverse: 5′-CAGCCCATAACAGCAGATAGGC-3′; GAPDH, F: 5′-CACCATTGGCAATGAGCGGTTC-3′, reverse: 5′-AGGTCTTTGCGGATGTCCACGT-3′) on a quantitative PCR instrument (Roche, Germany). Gene expression was quantified using the 2^△△Ct^ method.

### Western blot analysis

BEAS-2B cells or human lung tissue samples were lysed using the protein isolation kit (#KGP250, KeyGEN BioTECH, Nanjing, China) or homogenized with lysis buffer containing phenylmethylsulphonyl fluoride on ice, followed by centrifugation at 12,000 rpm for 5 min. Protein concentrations were measured using a BCA quantification kit (Keygentec, Nanjing, Jiangsu, China). Proteins were separated on 10% SDS gel and transferred to a polyvinylidene fluoride membrane, followed by 1 h of blocking with 5% skim milk. The membrane was then incubated with anti-N-cadherin (1:100; ab18203; Abcam), anti-vimentin (1:5000; ab92547; Abcam), anti-α-SMA (1:500; ab7817; Abcam), anti-E-cadherin (1:1000; ab76055; Abcam), or anti-GAPDH (1:10,000; PAB36264; Bio swamp, Wuhan, Hubei, China) antibody overnight at 4 °C, followed by 3 washes with Tris-buffered saline containing 0.1% Tween 20 (TBST). The membrane was then incubated with horseradish peroxidase-conjugated secondary antibody (1:10,000; SAB43710 or SAB43711; Bio swamp) for 2 h at room temperature. After an additional three washes with TBST and the addition of exposure solution (Millipore, #WBKLS0500, USA), the protein bands were visualized using an enhanced chemiluminescence reagent (Life iLab, Shanghai, China) and analyzed using the Image J software (NIH, Bethesda, MD, USA).

### Statistical analysis

Statistical analyses were carried out using Prism 6.0 software (Graphpad, San Diego, CA, USA). Data are presented as mean ± standard deviation. Differences between categorical variables were compared using the χ^2^ test. Comparison among groups were conducted using analysis of variance (ANOVA), followed by the Student–Newman–Keuls test. The association between FAM13A expression and variables of interest was analyzed by multivariate Cox proportional hazards analysis. The correlation analysis between FAM13A expression level and FEV_1_/FVC%, FEV_1_%pre, PO_2_, PCO_2_, E-cadherin, and vimentin were analyzed by partial correlation analysis the patient's age was the control variable. A value of *P* < 0.05 was considered statistically significant.

## Results

### EMT and EMT inducer TGF-β1 are associated with small airway remolding in COPD patients independent of smoking status

To evaluate the correlation of small airway remodeling with COPD, we measured the remodeling markers in lung tissue surrounding the small airways of the patients. As shown in Additional file 1: Fig. S1A–C, the lung tissue samples of COPD patients exhibited remarkably increased small airway wall thickness, collagen fiber deposition, and the protein levels of collagen I, fibronectin, and MMP9, as compared with those of non-COPD patients. No significant differences were observed in these parameters between non-smokers and smokers with COPD. In non-COPD patients, we only observed significantly increased small airway wall thickness in the smokers than those in the non-smokers. These results suggest that COPD patients undergo small airway remodeling independent of smoking status.

Considering the crucial role of EMT in airway remodeling, we detected the EMT markers in the lung tissue samples from the patients. As shown in Additional file 1: Fig. S2A and S2B, compared with those of non-COPD patients, the lung tissue samples of COPD patients had significantly attenuated E-cadherin and ZO-1 protein levels and markedly enhanced α-SMA, vimentin, and N-cadherin protein levels. No significant differences were observed between the non-smokers and smokers with or without COPD. Similar results were observed in Western blot analysis, except for significant differences in α-SMA and N-cadherin levels among non-smokers and smokers without COPD (Additional file 1: Fig. S2C). These results suggest that EMT is activated in the small airways of COPD patients independent of smoking status.

Because TGF-β1 is a potent inducer of EMT and plays an important role in the pathogenesis of airway remodeling [26], we further detected TGF-β1 protein levels in the lung tissue samples from the patients. Additional file 1: Figure S2D shows that in non-smokers, the lung tissue samples of COPD patients had significantly upregulated TGF-β1 protein levels.

### FAM13A is upregulated in the small airway epithelium in COPD patients and correlates with COPD severity and EMT marker expression

**Fig. 1:**
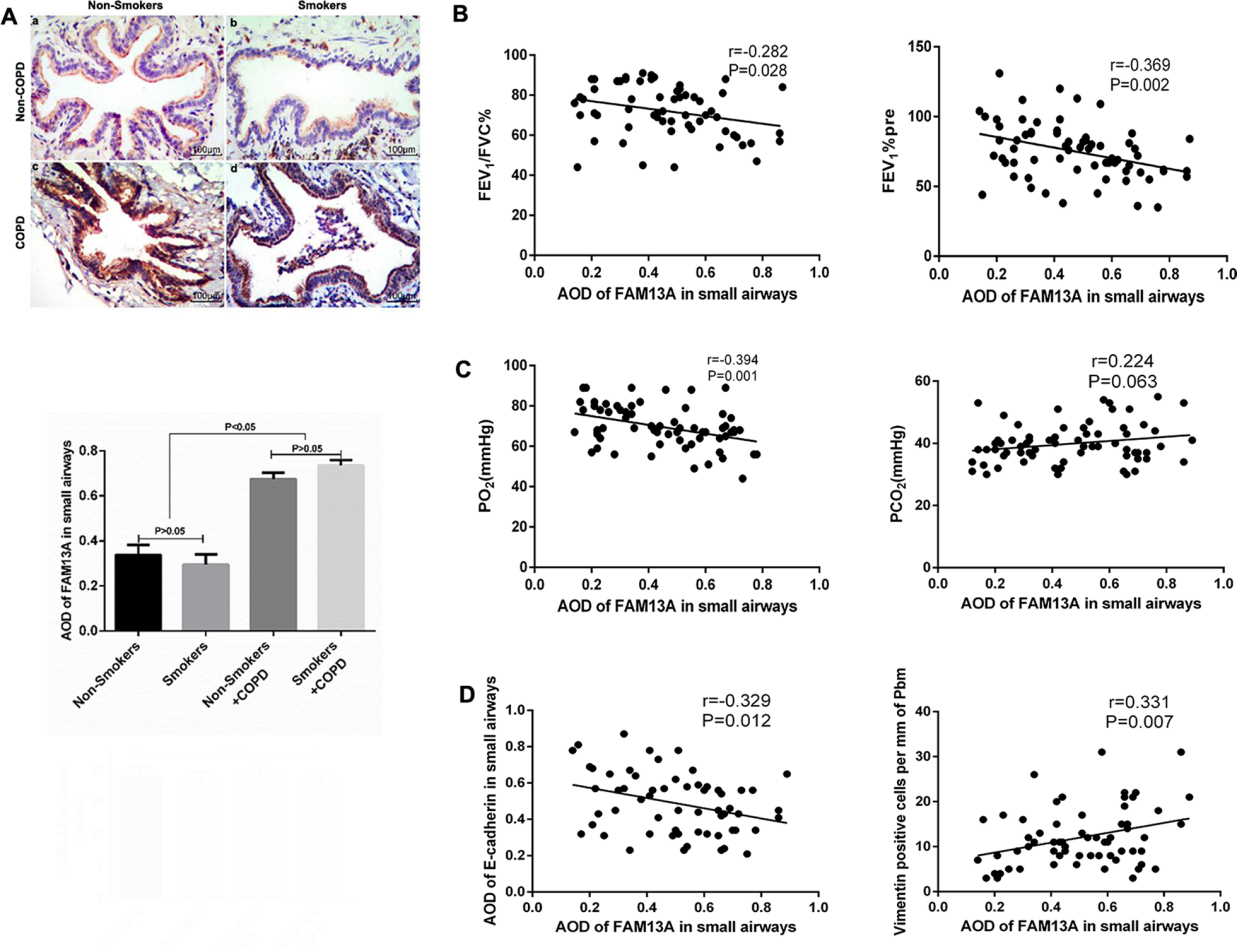
Expression of family with sequence similarity 13 member A (FAM13A) in small airway epithelium and its correlation with COPD severity and EMT marker expression. The patients were divided into non-smokers without COPD (n = 22), smokers without COPD (n = 23), non-smokers with COPD (n = 10), and smokers with COPD (n = 14) groups. **A** IHC staining was carried out to detect FAM13A protein expression in the small airway epithelium of the patients. **B**–**D** Multivariate Cox proportional hazards analysis, was performed to examine the correlations of FAM13A age as the control variable, expression with pre-bronchodilator FEV_1_%, PO_2_, PCO_2_, and expression of E-cadherin and vimentin. FEV_1_%pre: pre-bronchodilator forced expiratory volume in 1 s; PO_2_: partial pressure of oxygen; PCO_2_: partial pressure of carbon dioxide

To explore the involvement of FAM13A in small airway remodeling in COPD, we measured FAM13A protein levels in the small airway epithelium of the patients. IHC staining showed that FAM13A protein levels were considerably upregulated in the small airway epithelium of COPD patients independent of smoking status (Fig. 1A).

Then, we assessed the correlations of FAM13A protein levels with COPD severity. As shown in Fig. 1B and C, FAM13A protein levels in the small airway epithelium were significantly and negatively correlated with FEV_1_/FVC ratio, pre-bronchodilator FEV_1_, and PO_2_ of the patients. An opposite trend was observed in the correlation of FAM13A protein levels with PCO_2_, despite a statistical non-significance. These data suggest that FAM13A protein levels in the small airway epithelium are positively correlated with COPD severity. Furthermore, FAM13A protein expression was significantly and negatively correlated with E-cadherin protein expression, but positively correlated with vimentin expression (Fig. 1D), suggesting that FAM13A is associated with EMT in small airways.

### TGF-β1 stimulation upregulates FAM13A expression in BESA-2B cells

**Fig. 2:**
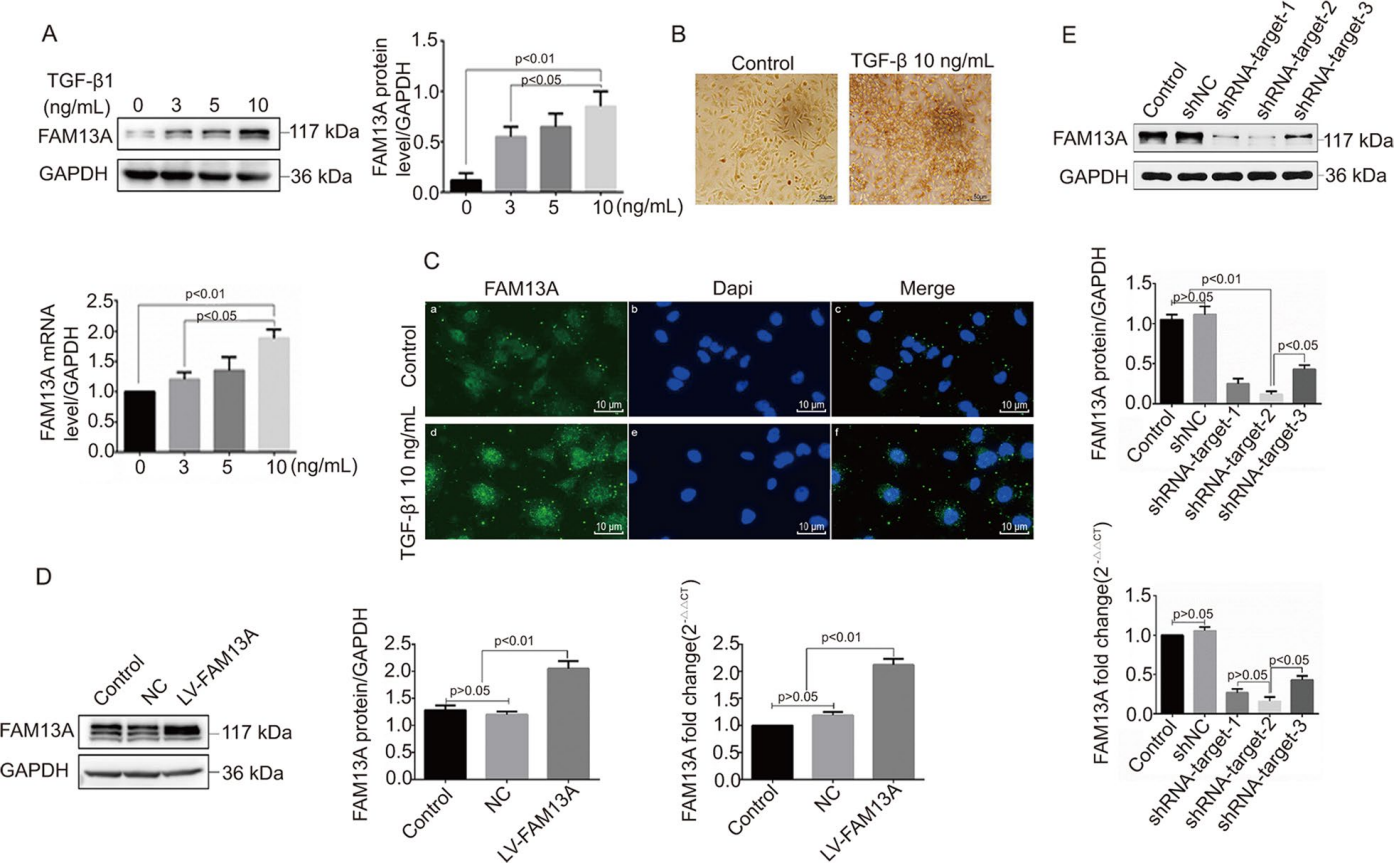
FAM13A expression in BEAS-2B cells stimulated by TGF-β1 and transfection efficiency of lentiviral vectors overexpressing FAM13A or small hairpin RNA (shRNA) against FAM13A. **A** BEAS-2B cells were stimulated with different concentrations of TGF-β1 (0, 3, 5, and 10 ng/mL) for 48 h. Western blot analysis was performed to measure FAM13A protein expression. **B** and **C** BEAS-2B cells were treated with 0 or 10 ng/mL TGF-β1 for 48 h. Immunocytochemical and immunofluorescent staining was conducted to detect FAM13A protein expression. Representative images are shown. Magnification × 200. **D** and **E** BEAS-2B cells were infected with lentiviral vectors expressing FAM13A or shRNA against FAM13A. Western blot analysis and quantitative real-time PCR were performed to measure protein and mRNA levels of FAM13A at 48–72 h after transfection. shRNA-target 2 shows the highest knockdown efficiency. Data are expressed as mean ± SD. n = 3 (technical replicates). The experiment was repeated three times

To understand the mechanism underlying the involvement of FAM13A in lung EMT, we determined FAM13A protein expression in BEAS-2B cells in response to TGF-β1 stimulation. As a result, TGF-β1 stimulation significantly enhanced FAM13A protein expression in a dose-dependent manner (Fig. 2A) in BEAS-2B cells. Immunocytochemical (Fig. 2B) and immunofluorescent (Fig. 2C) staining demonstrated consistent results. This finding suggests that FAM13A might be a downstream effector of TGF-β1 in the regulation of EMT.

### FAM13A promotes cell proliferation, migration, and invasion and is required for suppressing apoptosis in BEAS-2B cells

**Fig. 3:**
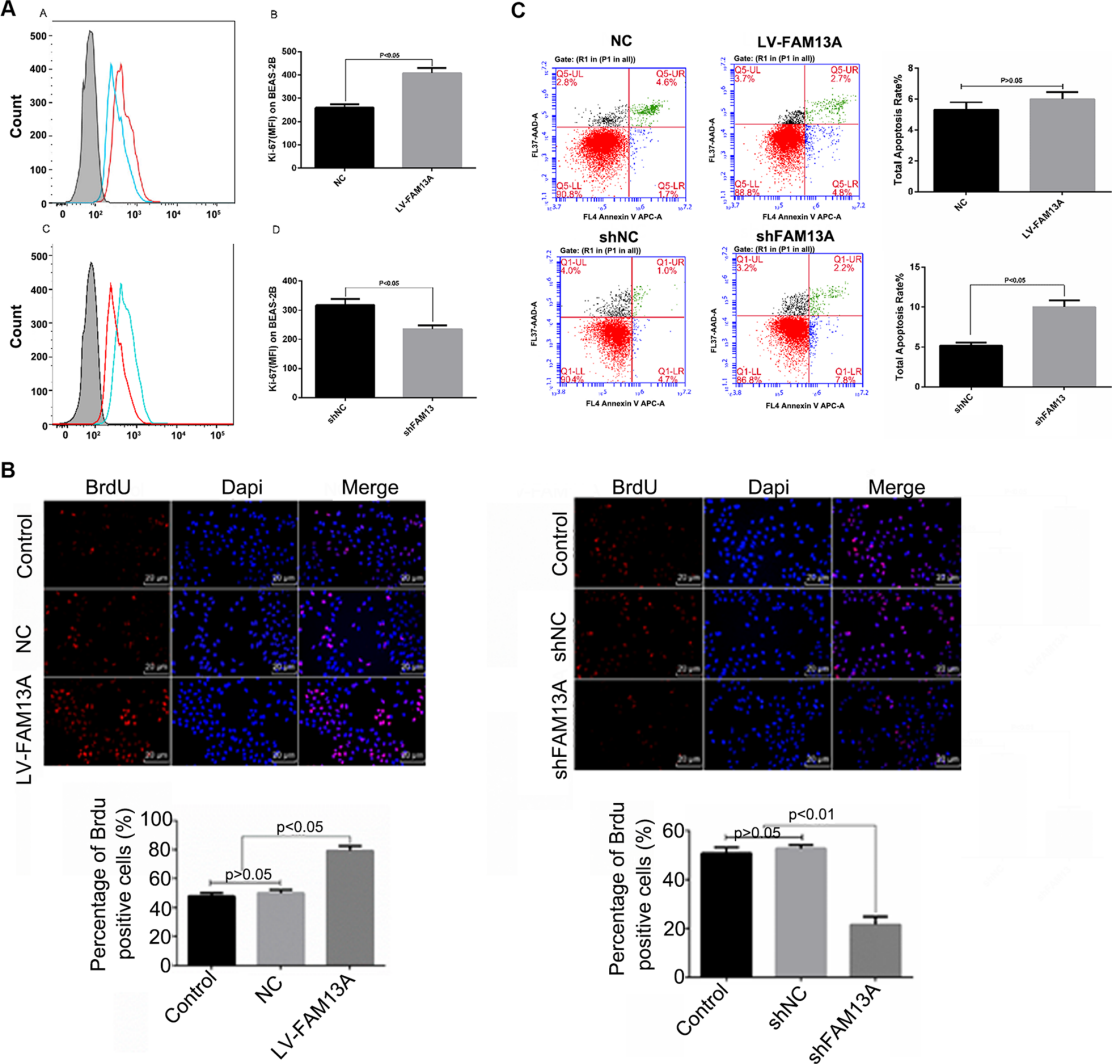
FAM13A promoted BEAS-2B cell proliferation. BEAS-2B cells were transfected with lentiviral vectors expressing FAM13A, shRNA against FAM13A, or corresponding negative control and incubated for 48–72 h. **A** Cells were stained with Ki67. Ki67-positive cells were detected by flow cytometry assay. **B** Cell numbers were stained with BrdU and observed under microscopy. Scale bar = 20 µm. 20 **C** Flow cytometry analysis was performed to examine cell apoptosis. Data are expressed as mean ± SD. n = 3 (technical replicates). The experiment was repeated three times

**Fig. 4:**
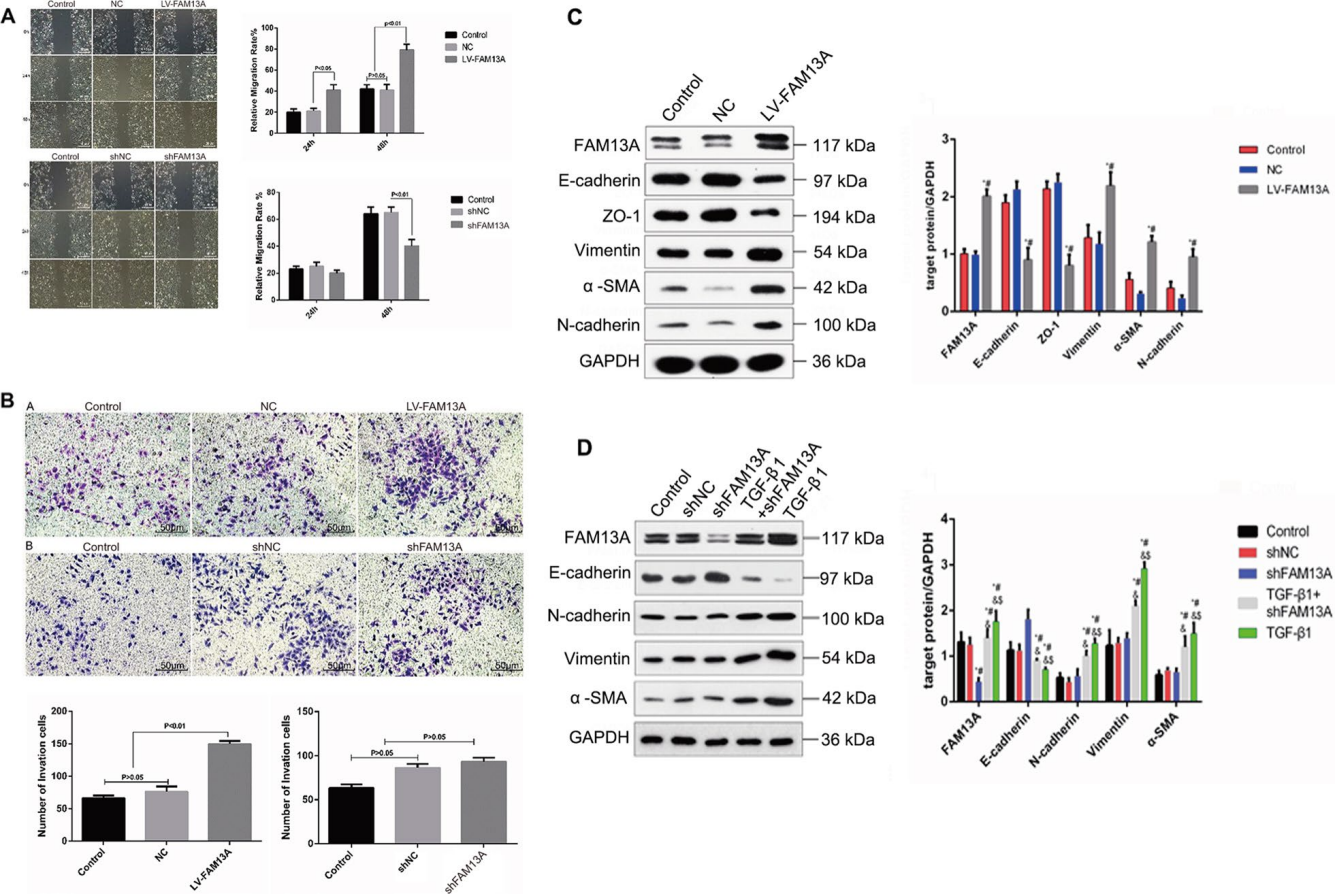
The effects of FAM13A on cell migration, invasion, and TGF-β1-induced EMT marker alterations in BEAS-2B cells. **A** and **B** BEAS-2B cells were transfected with lentiviral vectors expressing FAM13A, shRNA against FAM13A, or corresponding negative control and incubated for 48–72 h. Wound healing and Transwell chamber assays were performed to examine the roles of FAM13A in cell migration and invasion. Representative images are shown. Magnification × 100. **C** Upper panel: BEAS-2B cells were transfected with lentiviral vectors overexpressing FAM13A or negative control. Western blotting was performed at 48 h after transfection to measure the protein levels of FAM13A, E-cadherin, ZO-1, vimentin, α-SMA, and N-cadherin. GAPDH was used as an internal control. *P < 0.05 vs. control, ^#^P < 0.05 vs. negative control. Lower panel: BEAS-2B cells were transfected with shRNA against FAM13A or negative control. Cells were stimulated with TGF-β1 (10 ng/mL) for 48 h. Western blotting was performed at 48 h after transfection to measure the protein levels of FAM13A, E-cadherin, N-cadherin, vimentin, and α-SMA. GAPDH was used as an internal control. Data are expressed as mean ± SD. n = 3 (technical replicates). The experiment was repeated three times. NC: negative control; LV: lentiviral vectors. *P < 0.05 vs. control. ^#^P < 0.05 vs. negative control. ^&^P < 0.05 vs. shFAM13A. ^$^P < 0.05 vs. TGF-β1 + shFAM13A

Because epithelial cells acquire migratory and invasive properties during EMT [27], we sought to explore the functions of FAM13A in the proliferation and motility of BEAS-2B cells. Figure 2D and E show the transfection efficiency of lentiviral vectors overexpressing FAM13A or shFAM13A. Ki67 staining revealed that FAM13A overexpression significantly promoted cell proliferation, whereas FAM13A silencing significantly inhibited cell proliferation in BEAS-2B cells (Fig. 3A). Consistent results were observed by BrdU staining (Fig. 3B). In terms of cell apoptosis, although FAM13A overexpression failed to inhibit BEAS-2B cell apoptosis, FAM13A knockdown noticeably promoted BEAS-2B cell apoptosis (Fig. 3C). This finding suggests that FAM13A is required for the suppression of BEAS-2B cell apoptosis. In addition, FAM13A overexpression promoted cell migration and invasion, whereas FAM13A silencing inhibited cell motility in BEAS-2B cells (Fig. 4A and B). Taken together, these data suggest that FAM13A promotes the phenotype switch from epithelial to mesenchymal cells in airway epithelial cells.

### Knockdown of FAM13A abrogates TGF-β1-induced alterations in EMT marker protein expression in BEAS-2B cells

Next, we explored whether FAM13A is required for TGF-β1-induced EMT in airway epithelial cells. Western blotting revealed that FAM13A overexpression significantly attenuated E-cadherin and ZO-1 protein levels while enhancing vimentin, α-SMA, and N-cadherin protein levels in BEAS-2B cells (Fig. 4C). Although FAM13A knockdown did not affect EMT marker protein levels in BEAS-2B cells in the absence of TGF-β1, FAM13A knockdown partially reversed TGF-β1-induced alterations in the EMT marker protein levels in BEAS-2B cells (Fig. 4D). This finding suggests that TGF-β1 induces EMT in BEAS-2B cells at least partially via FAM13A.

## Discussion

Past clinical research has suggested that smoking is the most crucial risk factor for COPD. However, genetic factors also play an essential role in the susceptibility to COPD. In this study, we demonstrated that regardless of smoking status, the small airways of COPD patients exhibited active remodeling and EMT compared with non-COPD patients. EMT has been reported as an active process in the airways of COPD patients [28]. TGF-β1 induces EMT in the bronchial epithelial cells [29–31]. However, it remains unclear how TGF-β1 promotes EMT in small airway remodeling in COPD. This study shows that FAM13A can promote the proliferation, migration, and invasion of BEAS-2B cells, and knockout of FAM13A can partially reverse the changes in the expression of EMT markers induced by TGF-β1. These are consistent with previous reports concluding that FAM13A may be a potential EMT-correlated airway obstruction susceptibility gene [19, 32]. In their studies, FAM13A was demonstrated to increase the susceptibility to airway obstruction independent of smoking status [32] and to closely relate to the EMT of cystic fibrosis airway [19]. In summary, this study implies that FAM13A mediates TGF-β1-induced EMT in small airways in COPD, providing FAM13A as a potential therapeutic target for anti-EMT treatment of COPD.

Small airway remodeling in COPD involves fibroblast proliferation and migration, excessive collagen deposition, and an imbalance between ECM synthesis and degradation, resulting in airway wall thickening and luminal narrowing [6]. Increased deposition of ECM components, such as collagen fibers, fibronectin, α-SMA, and MMP-9, have been extensively used as airway remodeling markers by many clinical and experimental studies [33–35]. Our results showed that these remodeling markers were remarkably increased in the small airway epitheliums of COPD patients than non-COPD patients. Since EMT has been suggested as a core pathological factor in airway remodeling during COPD development [8, 9], we measured EMT marker expression in the small airway epithelium. As expected, the increases in the remodeling markers were accompanied by the upregulation of mesenchymal marker expression and downregulation of epithelial marker expression. These findings collectively indicate active remodeling and EMT processes in the small airways of COPD patients.

Interestingly, although studies have reported that smoking activates EMT and airway remodeling in COPD and other lung diseases [10, 36], our results showed that the alterations in these markers were independent of smoking status in COPD patients. This suggests that other factors contribute to EMT and airway remodeling, at least in COPD. Some investigators have demonstrated that in addition to environmental determinants, genetic risk factors, including genetic variants, epigenetic changes, and somatic mutations, may cause COPD [37–39]. Based on previous studies, including ours, demonstrating the associations of FAM13A with the increased risk of COPD in never-smokers and with lung EMT [19, 20, 22, 40], we sought to investigate the expression pattern and the roles of FAM13A in COPD. We found that the FAM13A protein level was elevated in the small airway epithelium in COPD patients compared with non-COPD patients, independent of smoking status, consistent with a previous report that FAM13A protein levels are elevated in very severe human COPD lungs [40]. In addition, we noticed that FAM13A protein expression negatively correlated with FEV_1_% and PO_2_ in COPD patients. This was consistent with a previous study that a FAM13A variant is associated with lower FEV_1_/FVC levels in never-smokers with COPD [32]. This finding suggests that high FAM13A expression is associated with severe COPD.

During COPD development, repetitive injury resulting from chronic exposure to environmental or genetic factors may cause persistent activation of repair processes in airway epithelium, such as EMT and altered proliferation and migration of bronchial epithelial cells [41]. In this study, FAM13A overexpression significantly promoted cell proliferation, migration, and invasion, whereas FAM13A silencing significantly inhibited these cell behaviors in BEAS-2B cells. Interestingly, it appears the regulation of FAM13A of cell proliferation is complicated and associated with environmental factors. Knockout of FAM13A in mice without exposure to smoke resulted in accelerated lung cell proliferation compared with FAM13A overexpression. Under the smoke condition, overexpression of FAM13A augmented the proliferation of alveolar septal cells [40]. In this study, we also attributed the proliferation of BEAS-2B cells to FAM13A and altering the expression of FAM13A induced the TGF-β1/ Smad signaling. Although in several in vitro studies, TGF-β1 has been demonstrated to be inhibitory to the growth of epithelial and tumor cells. Other reports support that TGF-β1 can confer bidirectional regulation of cell proliferation under different circumstances [42–44]. For instance, overexpression of TGF-β1 can cause the proliferation of epithelia in the head and neck, thereby promoting squamous cell carcinoma [43]. From our viewpoint, the involvement of TGF-β1/Smad in multiple signaling pathways cannot be ruled out, and how FAM13A functions and interact with TGF-β1/Smad signaling remains to be further elucidated. This will inspire us to perform future experiments with enlarged sample numbers, multivariable analyses, and in vivo approaches to increase knowledge of the molecular pathways.

Furthermore, FAM13A upregulation was implicated in small airway EMT, suggested by its significant correlations with attenuated E-cadherin expression and enhanced vimentin expression in the small airway epithelium. Hence, we speculated that FAM13A might facilitate COPD progression via EMT in small airways. These indicate that FAM13A is involved in the phenotype switch from epithelial to mesenchymal cells in airway epithelial cells. Despite its positive function in embryonic development and tissue repair, EMT has been extensively shown to play a key role in tumor formation and metastasis [45]. Recent studies also indicated that EMT might be vital to the pathogenesis of airway remodeling in COPD and other fibrotic diseases [46]. It can increase the number of fibroblasts and myofibroblasts in the airway wall and thus mediate airway remodeling. More than 50% of patients with COPD are accompanied by lung cancer [47, 48].

Furthermore, the expression of FAM13A is significantly increased in both COPD and lung cancer tissues [40, 49]. Some common mechanisms are likely shared by these two diseases [8, 12, 50, 51]. FAM13A has been reported as a downstream effector of EMT inducer TGF-β in A549 lung cancer cells [49]. Meanwhile, FAM13A is a susceptibility gene for lung cancer [52]. Its rs9224 site can change the susceptibility of squamous cell carcinoma by affecting the binding of miRNA-22-5p [53]. Our in vitro results showed that TGF-β1 stimulation upregulated FAM13A expression in BEAS-2B cells in a dose-dependent manner. In the meantime, TGF-β1 expression was significantly upregulated in lung tissue surrounding the small airways of COPD patients compared with non-COPD patients, independent of smoking status. These findings suggest a presence of TGF-β1/FAM13A signaling in the regulation of small airway EMT in COPD. Indeed, loss of FAM13A resulted in a partial but significant reversal of TGF-β1-induced alterations in EMT marker expression in BEAS-2B cells, suggesting that TGF-β1 regulates EMT in COPD airway remodeling at least partially via FAM13A. Previous studies have shown that FAM13A regulates RhoA activity through the rhoGAP domain, which causes changes in EMT markers of bronchial epithelial cells in lung cystic fibrosis [19]. In addition, in the pulmonary hypertension model, FAM13A can alleviate EMT of endothelial cells by inhibiting the β-catenin signaling of pulmonary artery endothelial cells [22]. As such, it will be of interest in a future study to find if the participation of FAM13A in the occurrence of EMT is a common mechanism linked with both COPD and lung cancer.

The limitation of this study is that even though two different pathologists determined the absence of lung cancer by observing the morphology of each section. However, due to unavailability, no specific marker was used to identify tumor cells.

## Conclusions

In conclusion, in this study, we demonstrated that FAM13A expression was upregulated in the small airway epithelium of COPD patients independent of smoking status. FAM13A promoted cell proliferation and motility and mediated TGF-β1-induced EMT marker alterations in BEAS-2B cells. Our results identify FAM13A as a novel mediator of TGF-β1 signaling in small airway remodeling, providing FAM13A as a potential therapeutic target for anti-EMT therapy against COPD.

## Supplementary Information

**Additional file 1.** Supplementary Figures.

## Data Availability

The datasets used and/or analyzed during the current study are available from the corresponding author on reasonable request.

## Abbreviations

FAM13A: Family with sequence similarity 13 member A
COPD: Chronic obstructive pulmonary disease
α-SMA: α-Smooth muscle actin
TGF-β1: Transforming growth factor-beta 1
shRNA: Small hairpin RNA
LV-FAM13A: Lentiviral vectors expressing FAM13A
PBS: Phosphate-buffered saline
