# Supplementary material for: Family with sequence similarity 13 member A mediates TGF-β1-induced EMT in small airway epithelium of patients with chronic obstructive pulmonary disease
Source: Respir Res. 2021 Jul 1;22:192. doi: 10.1186/s12931-021-01783-z (PMC8247231; doi:10.1186/s12931-021-01783-z)
Supplement: Supplementary file 1 — Additional file 1. Supplementary Figures. [file 12931_2021_1783_MOESM1_ESM.doc]

**Supplementary Figures**

**
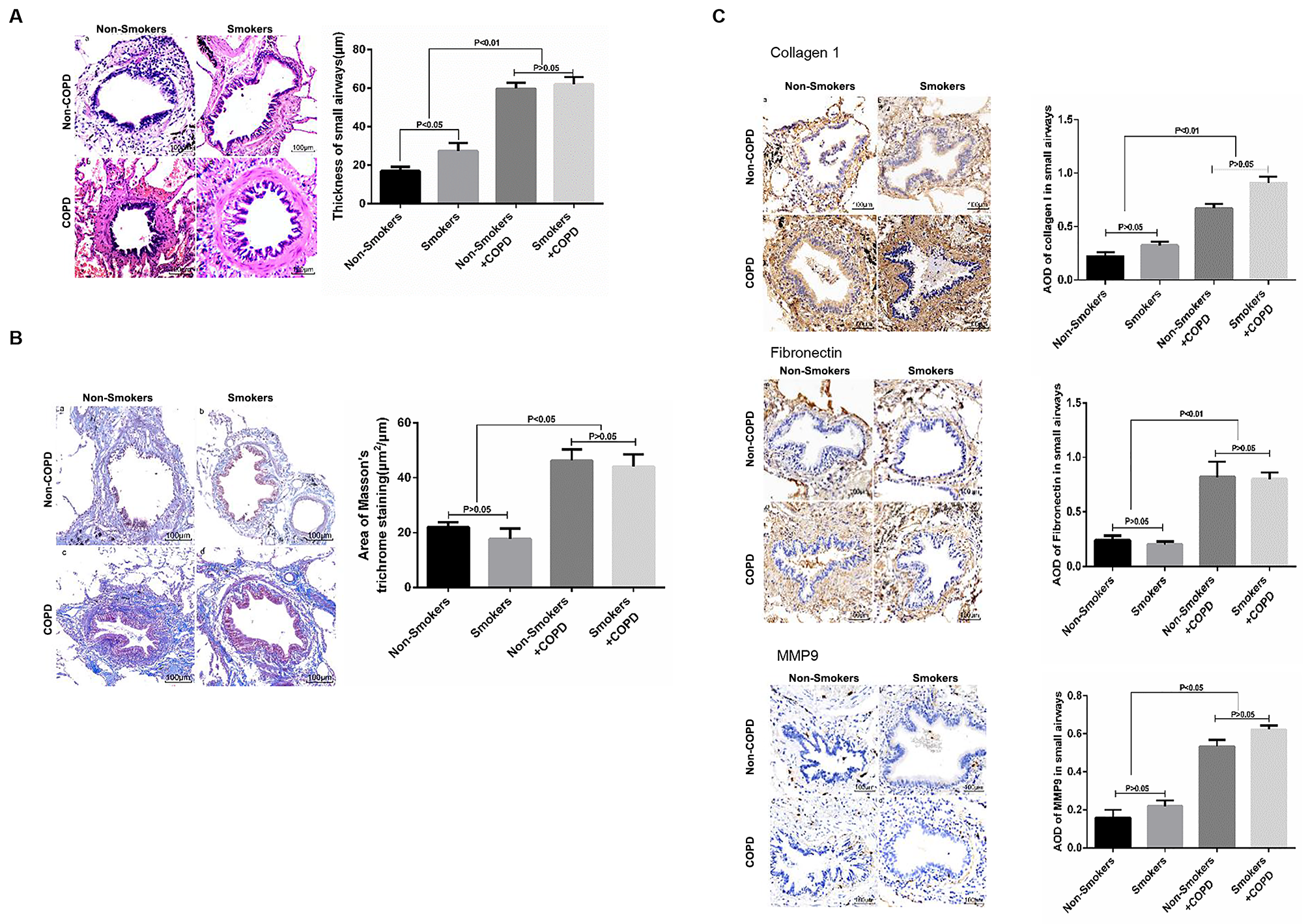
**

**Supplementary Figure S1. Examination of airway remodeling markers in lung tissue samples from patients with or without the chronic obstructive pulmonary disease (COPD).** Lung tissue samples were obtained from 35 patients with lung adenocarcinoma, 17 patients with lung squamous cell carcinoma, 12 patients with benign lung nodules, and 5 patients with bullous emphysema during lobectomy or lung volume reduction surgery. The patients were divided into non-smokers without COPD (n = 22), smokers without COPD (n = 23), non-smokers with COPD (n = 10), and smokers with COPD (n = 14) groups. **(A)** Hematoxylin and eosin (H&E) staining was performed. Small airway wall thickness was measured. **(B)** Masson’s trichrome staining was performed to measure the percentage of the collagen area. **(C)** Immunohistochemical (IHC) staining was conducted to determine the protein levels of collagen I, fibronectin, and MMP9. Representative images are shown. Magnification 200×. Scale bar = 50 µm. Data are expressed as mean ± standard deviation (SD). MMP9: matrix metallopeptidase 9; AOD: average optical density.


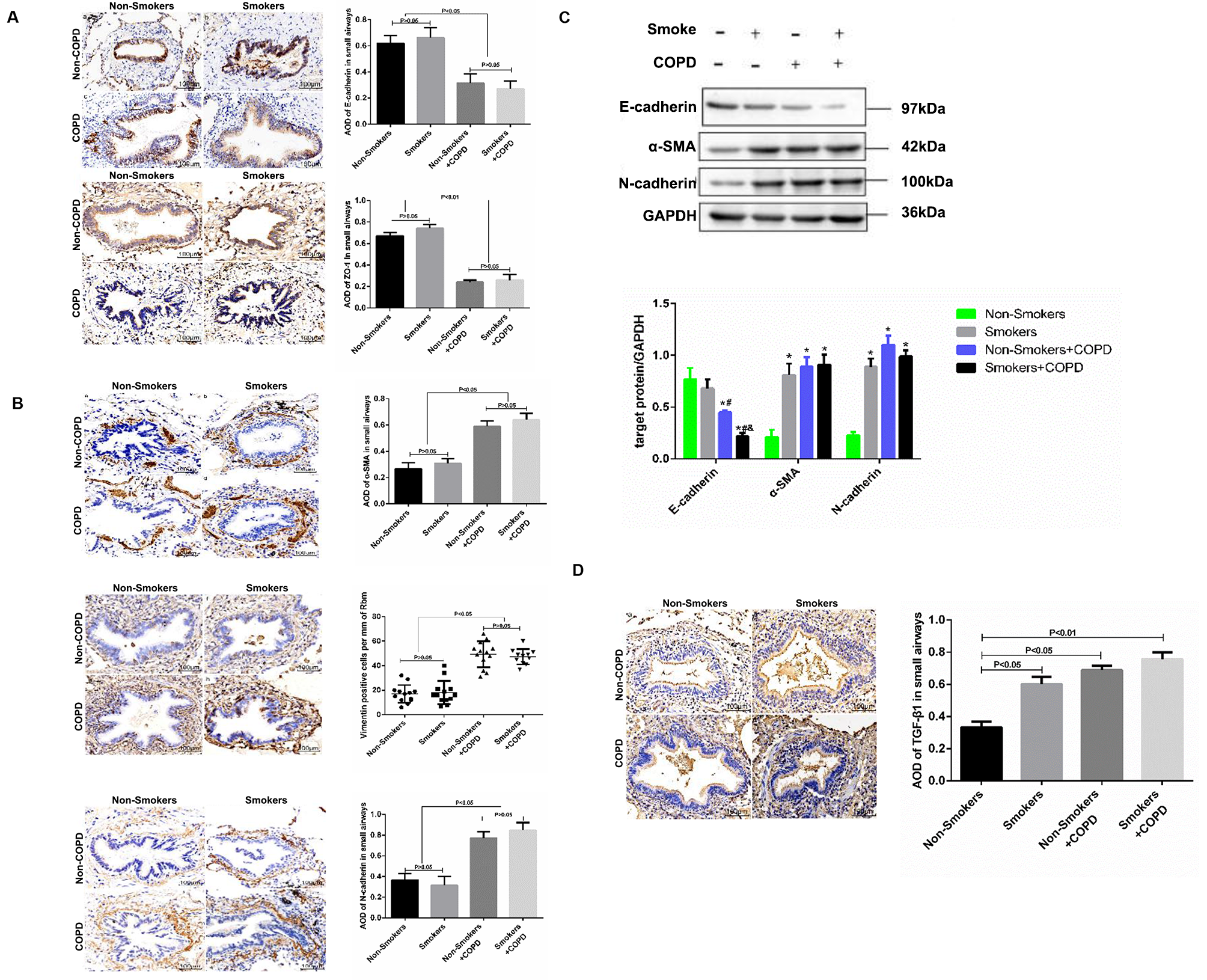


**Supplementary Figure S2. Expression of epithelial-mesenchymal transition (EMT) markers and** **transforming growth factor-beta 1 (TGF-β1) in lung tissue samples from the patients.** The patients were divided into non-smokers without COPD (n = 22), smokers without COPD (n = 23), non-smokers with COPD (n = 10), and smokers with COPD (n = 14) groups. **(A and B)** IHC staining was performed to determine protein expression of E-cadherin, ZO-1, α-SMA, vimentin, and N-cadherin in lung tissue surrounding small airways. **(C)** Western blot analysis was performed to measure protein levels of E-cadherin, α-SMA, and N-cadherin in lung tissue samples from the patients. GAPDH was used as an internal control. Data are expressed as mean ± SD. **(D)** IHC staining was performed to detect the protein expression of **TGF-β1** in lung tissue surrounding small airways**.** Representative images are shown. Magnification 200 ×. Scale bar = 50 µm.


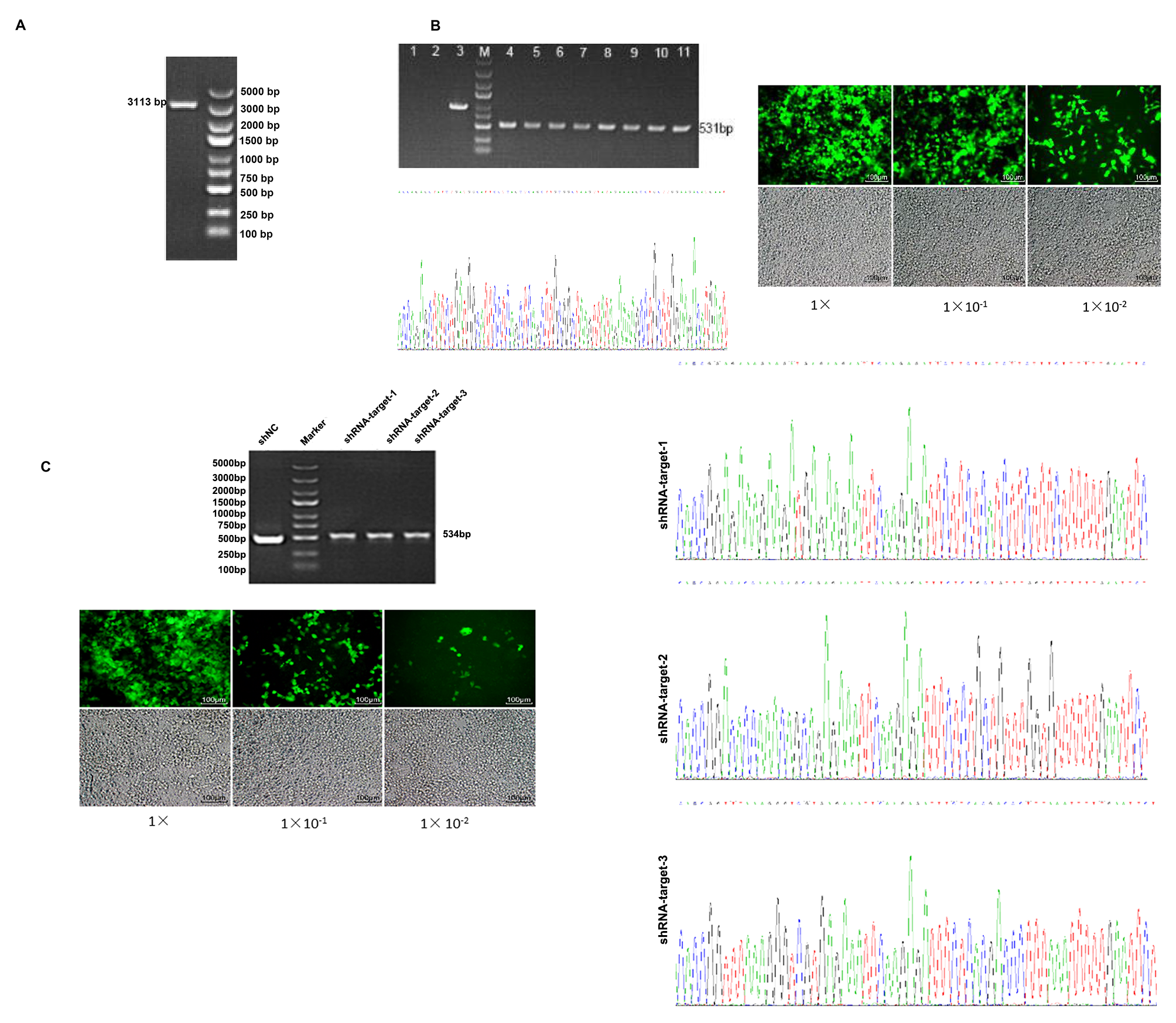


**Supplementary Figure S3.** **Construction of lentiviral vectors expressing** **FAM13A and shRNA against FAM13A.** **(A)** Agarose electrophoresis of digested coding sequence (CDS) of human FAM13A (3113 bp). **(B)** Agarose electrophoresis shows that FAM13A CDS was ligated into the lentiviral vector GV492. A part of the sequencing results are shown. HEK293T cells were transfected with the expression vectors. Green fluorescence was observed at 48 h after transfection under a fluorescence microscope at concentrations of 1×, 1×10^-1^, and 1×10^-2^, respectively. Scale bar = 100 µm. **(C)** Double-stranded DNA–coding shRNA-1, -2, or -3 for FAM13A was cloned into a lentiviral vector GV248. A part of the sequencing results are shown. HEK293T cells were transfected with the expression vectors. Green fluorescence was observed at 48 h after transfection under a fluorescence microscope at concentrations of 1×, 1×10^-1^, and 1×10^-2^, respectively. Scale bar = 100 µm.
